# Integrated Process Model Applications Linking Bioprocess Development to Quality by Design Milestones

**DOI:** 10.3390/bioengineering8110156

**Published:** 2021-10-24

**Authors:** Christopher Taylor, Lukas Marschall, Marco Kunzelmann, Michael Richter, Frederik Rudolph, Judith Vajda, Beate Presser, Thomas Zahel, Joey Studts, Christoph Herwig

**Affiliations:** 1 Koerber Pharma Software PAS-X Savvy, Mariahilfer Straße 88A/1/9, 1070 Vienna, Austria; christopher.taylor@werum.com (C.T.); lukas.marschall@werum.com (L.M.); thomas.zahel@werum.com (T.Z.); 2 Research Area Biochemical Engineering, Vienna University of Technology, Gumpendorferstrasse 1a, 1060 Vienna, Austria; 3 Boehringer Ingelheim Pharma GmbH & Co. KG, Birkendorfer Str. 65, 88397 Biberach an der Riss, Germany; marco.kunzelmann@boehringer-ingelheim.com (M.K.); michael.richter@boehringer-ingelheim.com (M.R.); frederik.rudolph@boehringer-ingelheim.com (F.R.); judith.vajda@boehringer-ingelheim.com (J.V.); beate.presser@boehringer-ingelheim.com (B.P.); joey.studts@boehringer-ingelheim.com (J.S.)

**Keywords:** digital twin, QbD, integrated process model, statistical modelling, bioprocess, control strategy, FMEA, severity rankings, development, risk assessment, DoE

## Abstract

Maximizing the value of each available data point in bioprocess development is essential in order to reduce the time-to-market, lower the number of expensive wet-lab experiments, and maximize process understanding. Advanced in silico methods are increasingly being investigated to accomplish these goals. Within this contribution, we propose a novel integrated process model procedure to maximize the use of development data to optimize the Stage 1 process validation work flow. We generate an integrated process model based on available data and apply two innovative Monte Carlo simulation-based parameter sensitivity analysis linearization techniques to automate two quality by design activities: determining risk assessment severity rankings and establishing preliminary control strategies for critical process parameters. These procedures are assessed in a case study for proof of concept on a candidate monoclonal antibody bioprocess after process development, but prior to process characterization. The evaluation was successful in returning results that were used to support Stage I process validation milestones and demonstrated the potential to reduce the investigated parameters by up to 24% in process characterization, while simultaneously setting up a strategy for iterative updates of risk assessments and process controls throughout the process life-cycle to ensure a robust and efficient drug supply.

## 1. Introduction

State-of-the-art bioprocess development seeks a balance between reducing time to market, while satisfying the regulatory requirements as described by the American Food and Drug Administration’s proposed validation cycle [1]. These guidelines require extensive process understanding to provide sufficient control in order to ensure that later process changes, whether for scale-up, optimization, or trouble-shooting, do not lead to substantially different product attributes [2]. Generating sufficient data to ensure this process control in situations of potentially unknown variability requires significant experimental resources and time.

**Figure 1:**
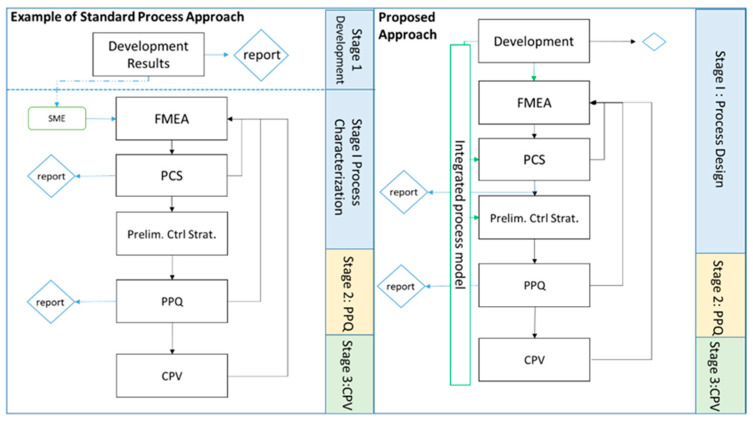
Example of a standard characterization workflow (**left**) vs. the proposed IPM-based workflow (**right**). Three aspects are of note. First, an algorithmic link between development and PCS can iteratively add data to an integrated process model. Second, the baseline FMEA severity ranking and preliminary control strategy are statistically underpinned. Third, since the FMEA and preliminary control strategy results are automatically generated in parallel, it removes linear effort in task completion. These activities then supplement the process performance qualification (PPQ) and continued process verification (CPV) stages of validation.

An emphasis on the implementation of models as early as possible in process development could potentially save constrained resources and maximize process understanding. In silico process models can also create a baseline upon which late-phase characterization is augmented, ensuring that all data is used at all stages of development, and that all data generated supports process knowledge and control at the most critical steps. Additionally, connecting these potentially separate data sources and models would enable the iterative improvement of holistic process understanding as described by quality by design (QbD, Figure 1) [3]. For ease of terminology, development data refers here to all data generated prior to process characterization studies (PCSs).

In particular, two deliverables from the FDA QbD framework (i.e., Stage I Process Design) could be significantly supported by quantitatively leveraging models from development:

- Risk assessment, often in the form of a failure mode and effects analysis (FMEA), which are conducted by process experts in order to assess relationships between potential critical process parameters (pCPPs) and critical quality attributes (CQAs), which then are iteratively reinforced by subject matter expertise and experimental results;
- Preliminary control strategy establishment for the CPPs by determining the CPPs’ proven acceptable range (PAR) for all CQAs.

One promising technology in this field is the integrated process model—an in silico depiction of the entire process chain, where relatively sparse unit operation data can nonetheless be statistically fitted for use in an overarching model. The unit operations are modelled individually. These models are then connected by using the output (dependent variable) of one model as input (independent variable) for the subsequent model, forming the integrated process model [4]. Critically for bioprocesses, the relationships between the unit operations are to be depicted in the overall model so that these applications’ predictions can be propagated out to drug substance, rather than being limited to the individual unit operation. That is, the impact of even the first unit operation’s process parameters can be modelled in silico onto the CQAs at drug substance.

Within this collaboration, we present novel IPM applications, leveraging only existing pre-process characterization data to (1) algorithmically establish risk assessment severity rankings, thereby determining CPPs, while also (2) generating PARs for those CPPs. The presented approach provides a concrete link between existing development data and process characterization, and potentially removes subjectivity where data-driven conclusions are otherwise challenging.

### 1.1. Risk Assessment Severity

Risk assessments such as the FMEA are perhaps the QbD deliverable most sensitive to the quality and quantity of available data, as they must overcome certain numerical [5] and psychological challenges [6]. Specifically, critical impact or severity on a CQA by a process parameter (which leads to the CPP designation) is determined through discussion and process expertise within the perspective of a single unit operation [7]. Rankings are revisited iteratively throughout the product life cycle.

Inclusion of all available data in the risk assessment via models creates the benefit of a more quantitative approach to process understanding. A number of complex procedures to quantify these rankings have been proposed and discussed in recent years [8,9,10]. While all of these methods contribute to the improved modelling of risk, these methods generally do not include the link between each of the unit operations in order to connect conclusions to the final impact on drug substance.

Thus, we seek to establish a quantitative and automated determination of the FMEA severity rankings, based on a model of the available data, that assesses all pCPPs’ impact on drug substance, irrespective of that parameter’s unit operation, by propagating impact through the entire process flow. We then look to algorithmically assign the severity ranking via a linearization of modelled pCPP impact on the drug substance out-of-acceptance (OOA) likelihood against a predefined critical limit.

### 1.2. Preliminary Control Strategy

The control strategy is the final manufacturing goal after designating a parameter as a CPP and is typically preliminarily established before process validation Stage II (Process Performance Qualification). A control strategy is any system designed to ensure that CPPs remain in a constant state of control that is required to ensure quality during manufacturing [11]. An essential metric for control strategies is the proven acceptable range (PAR): the range for a CPP within which all CQAs consistently remain within acceptance criteria, while all other parameters stay at set point or within normal operating variation [12].

Djuris provides an overview of available tools and techniques used to achieve this milestone [13]. Most of the approaches used for classifying pCPPs into critical and non-critical designations compare them by their potential impact on quality attributes and the likelihood to cause a result exceeding predefined limits [14,15]. The determination of control ranges is often made based on statistical models, where the limits are defined for the input parameters in such a way that the response variables meet predefined limits [16]. In assessments of process repeatability, parameters to be monitored are also compared against predefined acceptable ranges [17].

All these approaches have in common that they are highly dependent on which quality attribute results are acceptable. For the last unit operation before drug substance, specification limits (if available) can be directly applied; however, for intermediate process steps the assignment of acceptable ranges becomes a challenge, as it is not known which quality attribute levels are acceptable. An improvement would therefore be to use linked data across all unit operations to determine all intermediate ranges.

Eon Duval aims to solve this by linking the statistical models of individual unit operations, feeding the output of the previous unit operation as load into the subsequent unit operation [18]. As input concentrations, they use the worst-case prediction within the normal operating range. This approach has some drawbacks:

- For each CPP, there exists a worst-case condition for each response. The worst case for CQA 1 might not be the worst case for CQA 2.
- The worst case is not the most likely condition. Processes are usually performed at set point conditions and the normal operating ranges represent the uncontrollable variation, meaning that the most probable condition is the set point.
- For the uncertainty of the model predictions, the lower or upper 95% confidence interval is used as a worst case. This does not take into account that the most likely prediction is the model mean at set point.
- The models are only based on small-scale data and manufacturing data is not considered.

Peterson pointed out that for setting up control ranges (i.e., design space), uncertainty in the model prediction needs to be taken into account [19]. Additionally, the uncertainty around the process parameters (i.e., normal operating range) needs to be taken into account as well [20].

To our knowledge, the IPM covers all of the aspects discussed above [21]. We aim to leverage this methodology to set up a control strategy that considers the linkage between unit operations and the uncertainty around process parameter set points [21].

In this collaboration, we will present the following applied techniques with the above goals in mind:

- Create an IPM by concatenating development generated statistical models, thereby establishing an in silico version of the process [21].
- Assess risk assessment severity rankings by application of an IPM parameter sensitivity analysis and rank linearization algorithm that quantifies the behavior of each parameter with regard to its OOA probability, and compares this against a predefined critical OOA rate, assigning an FMEA severity ranking based on the impact at drug substance.
- Propose PAR limits per CPP and CQA by detecting increases in simulated drug substance OOA results across the CPP screening range and assigning a cut-off representative of a predefined acceptable OOA probability.

Finally, we present the above as a proof-of-concept case study using a candidate monoclonal antibody process provided by Boehringer Ingelheim to assess the results of the above procedures.

## 2. Materials and Methods

### 2.1. Candidate Process for Case Study

For the applications in this case study, in a collaboration with Boehringer Ingelheim in Biberach, Germany, data sets stemming from development and pre-PCS studies were made available for a candidate monoclonal antibody that depicts a potential platform bioprocess. For this process, the following aspects were relevant to the IPM:

- Eight downstream unit operations, consisting of a capture step (CAP), an acid treatment step (AT), an anion exchange chromatography step (AEX), and a cation exchange chromatography step (CEX), for which process development activities were carried-out. Additional data exists at set point for the following unit operations: depth filtration (DF), ultra-diafiltration (UFDF), viral filtration (VF), and the resulting drug substance (DS).
- DoE-based ordinary least square models, which were fitted, discussed, and selected with subject matter experts before inclusion in the IPM. The experiments were carried out in small-scale systems representative of the manufacturing scale. Subject matter experts evaluated the suitability of these systems prior to experimentation. Model variable selection was based on a standard procedure of selecting the model with lowest Akaike information criterion, and the following diagnostics were then assessed for model significance: R^2^_adj_, Q^2^, RMSE, and partial *p*-values, as well as the model residuals. All selected models were then discussed with the process experts for process plausibility before acceptance. Acceptable regression models were found for the unit operations CAP, AT, AEX, and CEX.
- Manufacturing data for specific clearance models and yield/clearance calculations as required for the unit operation linkage described elsewhere for all unit operations from two industrial scales [21]:
  ○ Two manufacturing-scale runs;
  ○ Three pilot-scale runs.
- Four CQAs typical of monoclonal antibody products, depicting three impurities (CQA1_imp_, CQA2_imp_, CQA3_imp_) and one desired product attribute (CQA1_prod_):
  ○ Each of the above CQAs has an acceptance limit at drug substance in place.

### 2.2. Data for the Integrated Process Model

The IPM technology used here is described in detail elsewhere [21]. Regression models that depict the performance of a unit operation as a function of its process parameters were fitted to the four described responses’ specific clearance (*SC*). Briefly, specific clearance is used here as a general term for the specific, non-volumetric increase or decrease of a CQA, although a desirable product trait may also be accurately described as yield. This term enables a cross-unit operation transfer of the output units as seen in Equation (1) below:

(1) $$SC=PP\cdot \beta_{PP}+\beta_{0}+\varepsilon$$

where *SC* is a vector of the measured specific clearances, *PP* is a (*n* * *p*) matrix of the process parameter settings of each DoE run, *β_PP_* is the regression coefficient, and *β_0_* is the intercept. These models will be referred to as DoE models in the following.

Additionally, unifactorial regression models describing the specific clearance performance of a unit operation as a function of the specific load concentration (*SLC* in Equation (2)) onto pool specific concentration were calculated similarly, providing the link between unit operations. These models will be referred to as load models in the following.

(2) $$SC=SLC\cdot \beta_{SLC}+\beta_{0}+\varepsilon$$

For cases where a DoE model and a load model were both available for a unit operation, the results of the prediction were combined according to Equation (3). The predicted clearance at the sampled process parameter settings was corrected by the change in clearance due to a change in *SLC*.

(3) $${\hat{SC}}_{i}=\hat{SC}\left(PP_{i}\right)\;\cdot \frac{\hat{SC}\left(SLC_{i}\right)}{\hat{SC}\left(\bar{SLC_{DoE}}\right)}$$

where $\hat{SC}\left(PP_{i}\right)$ is the expected clearance at the process parameter settings for the current simulation cycle, $\hat{SC}\left(\bar{SLC_{DoE}}\right)$ is the expected clearance at the mean *SLC* from the DoE runs, and $\hat{SC}\left(SLC_{i}\right)$ is the expected clearance at the *SLC* of the current simulation cycle.

The starting concentration for each CQA was assumed to be normally distributed. The mean and standard deviation were estimated from manufacturing-scale runs.

For the case study described, all identified and applied models are summarized as a heat map in Table 1 and again in greater detail in the supplemental information Tables S1 and S2. The underlying assumption is that during the subsequent simulations, sampled prediction variation will be depicted in proportion to the RMSE and should generally correspond to the standard deviation of any reproducible (i.e., set point) runs.

### 2.3. Parameter Sensitivity Analysis

The parameter sensitivity analysis (PSA) is a specialized application of the IPM Monte Carlo simulation [21]. The PSA assesses how the change of each parameter across the full investigated screening range influences OOA events at drug substance. For each process parameter per unit operation for which there was a statistical model, the PSA was conducted per the following procedure:

The individual process parameter’s experimental screening range was divided into 10 equidistant points, referred to as grids, with 10 being the grid size for this study.

At start of the simulation, the parameter was fixed at the grid point at the lowest end of the screening range.

All other process parameters were allowed to vary around their set point within the described normal operating ranges. The process parameters were assumed to be normally distributed with the set point being the mean and the normal operating range being ±3 standard deviations.

The full Monte Carlo simulation was performed 1000 times at the above conditions for each CQA. An average *OOA* (%) result was recorded for each CQA, based on a predetermined acceptance limit.

The individual CPP was then fixed to the next grid and the cycle was re-performed.

Once all grids in the grid size were simulated, the %*OOA* result was plotted across the screening range.

*OOA* probability per CQA was calculated according to Equations (4) and (5) below. While the calculation of *OOA* percent likelihood could further be optimized to include non-parametric procedures for the selected statistical models, the normality assumption at the drug substance level largely applies.

(4) $$OOA=P\left(X\leq Lower\;Spec\;Limit\;\cup^{\;}X\geq Upper\;Spec\;Limit\right)$$

where *X* is a normal random variable, $N\left(\bar{x},s^{{*}^{2}}\right)$, where s* is the upper one-sided confidence limit of the standard deviation.

(5) $$s^{*}=s\cdot \sqrt{\frac{n-1}{\chi^{2}\left(\gamma ,n-1\right)}}$$

The upper confidence interval of the standard deviation was used as an estimate of precision in order to allow for a fair comparison between the *OOA* rate between observed large-scale data and in silico runs. The sample size of the available large-scale data was very small compared to the runs generated in silico.

PSA results can be plotted in various ways. The individual CQA results can be plotted against an overlay of all CPPs with scaled and centered parameter screening ranges, with the goal of rapidly determining the most impacting parameters. Conversely, the results can be plotted per CPP against all CQAs. The CPP-based plot is useful for determining a control strategy (described in the next chapter), while the CQA plot allows for quick interpretation of focal points for each CQA during the process. The suggested procedure would be to first identify CQAs strongly impacted by certain CPPs (i.e., plot by CQA) and then to drill down into the CPP-based plots.

Here, we additionally describe an innovative plotting overlay for all CPPs per CQA, referred to as ‘relative screening range’: a plot of a combination of standard data coding, but fixed to the manufacturing set point. The rescaling of the data is generally standardized on a −1,1 scale, but in this case it was additionally allowed to shift such that the set point always represented 0. This implies that in the most extreme case where the upper/lower limit of the screening range also represented the set point, the screening range was coded −2 to 0 (or 0,2). Equation (6) is as follows for each point in the screening range:

(6) $$x_{i}\prime =\frac{x_{i}-x_{sp}}{\frac{x_{max}-x_{min}}{2}}$$

where *x_i_*^′^ is the rescaled value of the ith value of parameter *x*, *x_i_* is the original ith value of parameter *x*, *x_sp_* is the manufacturing set point of the parameter corresponding to *x*, and *x_min_* and *x_max_* are the minimum and maximum points of the screening range for parameter *x*.

This not only makes it possible to quickly see the magnitude with which parameters impact any number of CQAs, but also to see where the set point lies with regard to the explored range, giving an indication of where there may be room for the restriction or expansion of parameter ranges.

## 3. Results

The development process data provided by Boehringer Ingelheim were successfully used to create an IPM consisting of specific clearance and multi-linear regression models for all four CQAs, to be simulated across seven unit operations.

To implement the proposed novel procedure, the following activities were executed, as described below: (1) IPM plausibility check and confirmation as an adequate model collection, (2) automated generation of risk assessment severity rankings leading to CPP designation, and (3) generation of the preliminary control strategy PAR settings for the CPPs.

### 3.1. IPM Plausibility Check

The quality of each OLS model contained and concatenated within the IPM was previously individually assessed based on R^2^_adj_, Q^2^, RMSE, *p*-values, and residual analysis. The linking of the models and the results of the Monte Carlo simulation were additionally checked visually by 1000 cycles executed under the manufacturing conditions (i.e., set point for all parameters with sampling from a normal distribution around the set point). A check of the actual data to the simulated data was performed, and the predicted in silico *OOA* results were compared to the observed *OOA* results.

**Figure 2:**
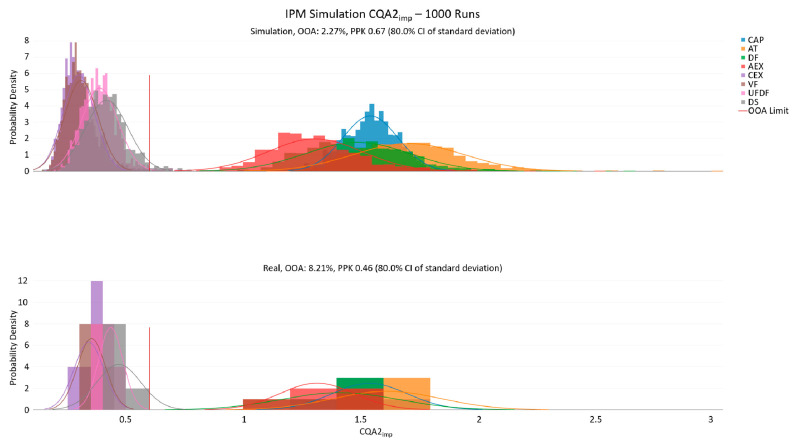
IPM trending plot for 1000 simulations (upper subplot) compared to real data performed (lower subplot) for a CQA impurity. The unit operation order is in descending order in the legend. One can clearly see the descent towards the final drug substance result. The simulation distributions align well with the sparse manufacturing data and the predicted *OOA* is slightly less than the predicted *OOA* incidence based on a normal distribution around the real manufacturing data.

Given the sparsity of manufacturing data at this stage of development, the simulated data corresponded adequately well to the existing manufacturing runs, and this repository of data can be seen as a baseline data set that may be used to proceed with further IPM applications (see example in Figure 2 below; remaining plots in the supplementary material, Figures S1–S3).

### 3.2. Automated Generation of FMEA Severity Rankings

Upon completion of the PSA simulation, the results were used for the FMEA severity ranking linearization. This procedure describes the ratio of the slope of simulated CQA OOA results to a predefined critical frequency of *OOA* results. A critical effect slope is defined here as the maximum allowable effect of the pCPP on the CQA between the manufacturing set point and the edge of the screening range (in units %*OOA*).

#### 3.2.1. Critical Effect Slope Determination

First, the critical effect is depicted as the slope between half the screening range (i.e., set point to the edge of screening range) and the intersection with a predefined critical frequency of *OOA* results. Here a limit of 5% allowable *OOA* results was chosen, corresponding to a population outside 2 standard deviations of the normally distributed results for a given CQA. This 5% limit was determined with the subject matter experts and the underlying risk management system.

**Figure 3:**
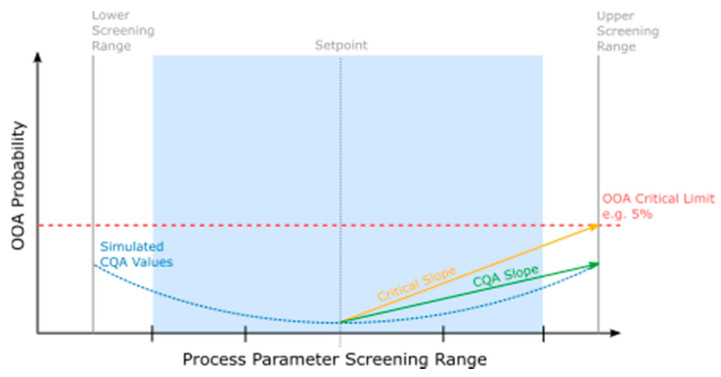
Example of FMEA linearization procedure. The shaded area represents the currently proposed manufacturing range.

Specifically, starting from the mean simulated value at the manufacturing set point, out to the intersection of the screening range (*x*-axis) and the critical effect (5% *OOA*, *y*-axis), a line was fit and subsequent slope was calculated. This was performed twice, on both sides of the manufacturing set point. Once established, these lines represent the maximum allowable severity of the pCPP impact on a CQA. These slopes are hereafter referred to as the ‘critical slopes’ (see Figure 3 as well as Equation (7)).

(7) $$m_{critical\;effect}=\frac{OOA_{SR,5\%}-OOA_{SP}}{CPP_{SR}-CPP_{SP}\;}$$

where *m_critical effect_* is the critical slope, *OOA* is the out-of-acceptance result as a percentage, *SR* is the screening range limit (max or min), *SR*,5% is the intersection of the screening range and 5% *OOA*, and *SP* is the set point.

#### 3.2.2. CQA Slope Determination

The CQA slope was fit in a similar manner, but based only on the simulated data for the CQA in question (see Figure 3). A line was fit between the results of the simulation at the set point value and the simulated mean value at the screening range limit. This slope was also calculated and repeated for both sides of the set point. Once established, these slopes represent the simulated relationship between the pCPP and the CQA in the *OOA* results. This slope’s relationship to the critical slope is essential to quantify how close it is to an unacceptable impact. These slopes are referred to as CQA slopes in Equation (8) below:

(8) $$m_{cqa}=\frac{OOA_{SR}-OOA_{SP}}{CPP_{SR}-CPP_{SP}\;}$$

where *m_cqa_* is the CQA slope, *OOA* is the out-of-acceptance result as a percentage, and *SP* is the set point.

#### 3.2.3. FMEA Ranking Algorithm

Once both the critical slope and the CQA slope were determined, the two slopes were compared as a ratio, with the critical slope being the reference slope and the CQA slope being calculated as a % reference value in Equation (9) below:

(9) $$\%ref=\frac{m_{cqa}}{m_{critical\;effect}}*100$$

The calculation was performed twice, once for each side of the screening range. The ‘worst-case’ slope of the two was chosen for the ranking. This %ref value was then compared to a classification rubric to determine the corresponding FMEA severity value (Table 2). As a baseline, any CQA slope equal to or larger than the critical slope must by design correspond to the highest severity ranking.

The FMEA ranking was determined using the agreed-upon rubric (Table 2), based on an internal company 10-point FMEA severity ranking scale. Other ranking scales may be used as well.

As a case study of the linearization methodology, the risk rankings of two selected process parameters that were already assessed in a current best-practices FMEA process (selected by process experts for proof of concept) were algorithmically assessed in the IPM and the results were compared.

**Figure 4:**
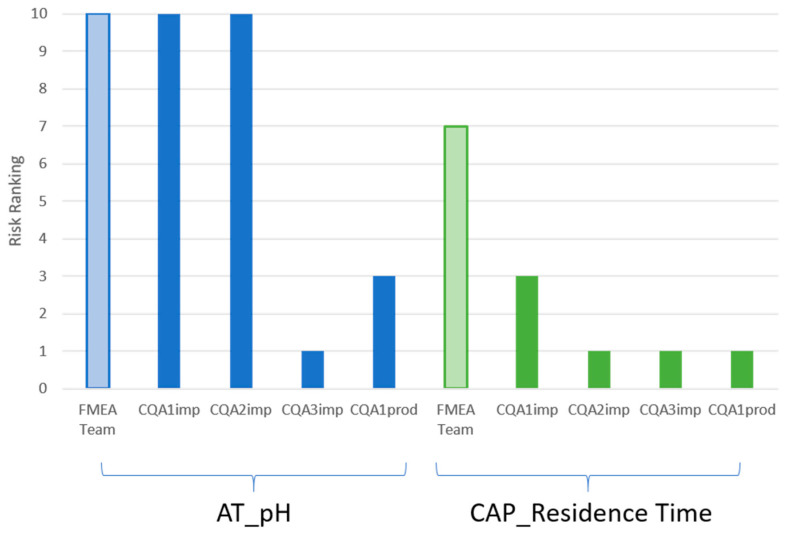
Results of the algorithmic setting of the FMEA severity ranking per CQA. The FMEA team bar represents the current process-expertise-based ranking with the granularity of final ranking for the worst-case CQAs. The IPM ranking assesses each of the CQAs against the 2 parameters in question. As CPPs are classified as ranking 10, AT_pH will be considered a CPP in both assessments, whereas CAP_Residence Time will be considered a PP. While both methods agree with the final ranking, there is a difference in the assessment of CAP_Residence Time, with the FMEA team ranking this as a more critical parameter than the PSA.

The risk rankings from the FMEA expert team and the risk rankings from the IPM application are compared in Figure 4. The results and rankings of the process-expertise-based FMEA evaluation were not made available before the completion of IPM in order to avoid any form of bias. Both the FMEA team and the IPM severity ranking agree that the parameter AT_pH should be considered critical. The IPM results also generally agree with the expert assessment of the CAP_Residence Time as a non-critical parameter.

#### 3.2.4. Preliminary Control Strategy Setting Procedure

In establishing the control strategy, the ICH Q8(R2) guideline provides a description of the proven acceptable range that could be translated to a quantitative description that may then be used to establish a control strategy using the PSA. According to the ICH, a proven acceptable range is ‘A characterized range of a process parameter for which operation within this range, while keeping other parameters constant, will result in producing a material meeting relevant quality criteria.’

We defined the PAR for each process parameter as the range where CQA results were within acceptance limits at drug substance at a certain probability level. Therefore, we first defined the critical level (i.e., the out-of-acceptance probability) that the manufacturer is willing to accept. Again, we defined this as 5% out-of-acceptance results (roughly equivalent to accepting 2 standard deviations of the population distribution).

**Figure 5:**
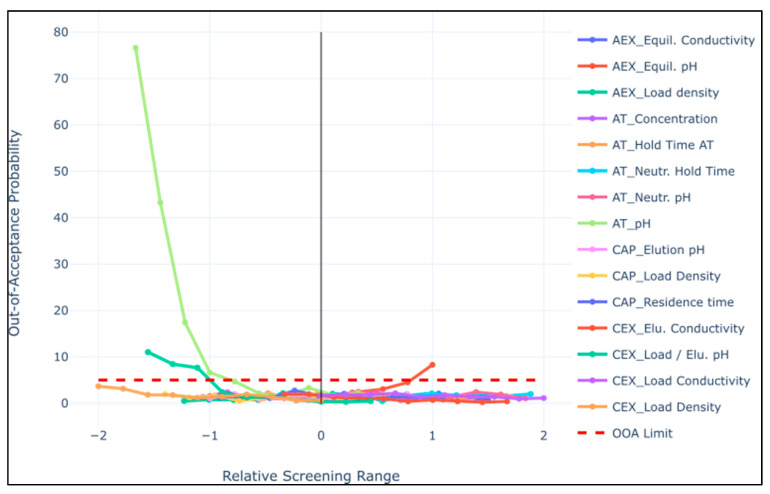
Relative screening range of CQA1_imp_ for all tested potential CPPs. The set point was coded to zero and the design space varied between −2 and 2 based on where the set point lies.

CQA OOA probability results are plotted across the screening range. Once the CQAs were identified as having high areas of risk, that is, the OOA >5% limit was crossed (see Figure 5), the PAR was set to the point at which 5% was intersected. This was iterated through all CQAs for all pCPPs and the most restrictive of the PARs was chosen as the ultimate PAR limit.

**Figure 6:**
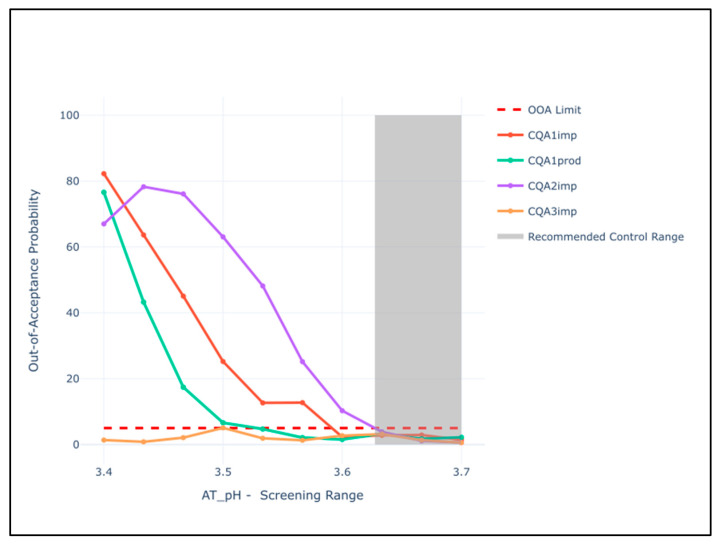
PSA plot for AT_pH. The impact of the 4 CQAs is depicted as a behavior across the screening range. The proposed control range is highlighted in grey. Note that AT is the second unit operation in process order, but the plots depict the impact on DS results.

The CPP-based PSA plots can be used for easy interpretation of the above procedure. In Figure 6, the behavior of all CQAs is shown across the full screening range for a given CPP. A line is drawn as each of the CQAs crosses the 5% threshold, and the final PAR is the most restrictive range. Lastly, areas of the screening range within PAR are in grey. Fifteen pCPPs were assessed in this manner. The control strategy plot for AT_pH is depicted below. Further plots can be found in the supplementary information per CPPs in Table 3 (Figures S4–S7).

Table 3 presents the generalized results for the PSA study, with regards to setting up a control strategy. The initially proposed manufacturing control range was either acceptable across the entire screening range, or only a subset of the original manufacturing control range was found to be acceptable; that is, a restriction on either higher or lower end is proposed. In this case, the impacted CQA is listed and plotted in the supplementary information (Figures S7–S9).

## 4. Discussion

The novel linkage of development data to the PCS QbD milestones of risk assessment severity ranking and preliminary control strategy establishment was proven feasible within the case study data provided by Boehringer Ingelheim. These models now improve characterization workflows by reducing FMEA subjectivity and decreasing required PCS resources as described below.

### 4.1. Automated FMEA Severity Ranking Linearization

In comparing the novel IPM FMEA result to the current best practice methods of FMEA ranking via process expertise, there are two aspects of the ranking that are of interest to compare:

− The overall FMEA severity ranking;
− The CQA(s) most likely to be impacted by the pCPP.

As can be seen in Figure 4, an identical overall CPP designation was generated between the IPM FMEA ranking and the current state-of-the-art experience-based FMEA ranking. Both evaluations assign critical and non-critical designations in the same manner for both process parameters. The FMEA team ranked the CAP_Residence Time as more critical than did the PSA algorithm, but both agreed that it should not be identified as a CPP. An advantage of the novel procedure is that it produced similar results, but is not dependent on long discussions or the availability of experts, nor is it subject to other challenges discussed in the introduction. Nonetheless, targeted discussions may be used if there is uncertainty about the PSA ranking, while still reducing the time consumed and focusing discussions on key findings.

The IPM method additionally generates risk rankings for all CQAs for each pCPP. Though this leads to the same ultimate conclusion as other methods (as in any case, the worst-case CQA ranking is taken as the final ranking), evaluating each process parameter with the individual CQAs has the advantage that it provides a more detailed and distinguished picture that adds to process knowledge. Here, it is of interest that two separate CQAs were severely impacted by AT_pH; this is considered to be a novel observation.

This granularity makes it so that, in the ensuing process characterization experiments, not all CQAs may need to be investigated at every unit operation, saving analytical costs. If a CPP is determined to not impact any investigated CQAs, it can be feasibly excluded from experiments in the PCS, reducing experimental cost. With the data-driven statistical evaluation of the parameters, there is a clear and easily describable justification for control of the parameter, and therefore the exclusion of the additional wet-lab work. Both provide a potentially substantial reduction in characterization effort.

Moreover, the IPM procedure provides a quantitative logic and result at the drug substance level that can be documented and visualized to demonstrate the data-driven impact of CPPs on CQAs. The ability to both quantify and visually display the full process relationships based on models significantly improves the understanding, justification of control, and ultimate acceptance by regulatory authorities in submission, while also removing some elements of subjectivity from risk-based decisions.

The analytical set here is small and therefore serves only as a proof of concept for the algorithm, and a full comparison of all investigated pCPPs would provide a better justification for the use of an automated severity risk assessment. Secondly, the IPM does not remove subjectivity completely. The screening range, as well as the predetermined critical limit (i.e., 5%), may include subjectivity.

Nonetheless, the ability to integrate the full process dataset into an automated ranking system provides improved detail, rationale, and less subjectivity, while potentially saving on future resources and increasing process control.

### 4.2. PSA Established Control Strategy

The IPM control strategy improves on current model-based PAR strategies by using a variation sampling technique that simulates the realities of manufacturing at scale, while concurrently establishing the PAR based on the results at drug substances, rather than at the individually modeled unit operations.

The simulation of the process parameters, sampled from within a normal distribution around the parameter set point, allows parameter settings to be simulated that are both more realistic (not simply worst case), and allows factors to interact in a multivariate space.

Furthermore, determining PAR on the results at drug substance also allows this variation to propagate throughout the process, delivering an accurate depiction of a process parameter’s impact on the final product.

One additional underlying benefit is that the technology used here is still based on standard ordinary least square models, which are well established and already being used in the determination of PAR settings (Burdick et al. 2017), thus reducing the effort required to make the logical case for the concatenation of the models in an IPM as well as the adequacy of the resulting proposed PAR limits (which are also later verified by the process performance qualification) in regulatory submissions.

Through the effective use of development data and the implementation of the models described here, a primary control strategy can be established, which will allow the development team to focus characterization studies only on areas where significant variability exists or on parameters that show a high level of uncertainty, thus allowing for clear justifications of control for all parameters and a reduced workload for process characterization—normally the largest work package for Stage I process validation.

This methodology is of course only as good as the models upon which it is built, and they are in turn dependent on the experimental conditions in development. As time and costs are a major constraint in pre-PCS studies, this could be a limitation to the efficacy of the proposed methods (e.g., screening designs are used instead of response surface designs). These results may nonetheless provide the basis for an iterative development road map, leading to the prioritization of targeted CQAs and CPPs in process characterization.

In this feasibility study, fifteen parameters were successfully modelled in the IPM, of which two were removed from later PCS studies. Thirteen remaining parameters that were then committed to PCS experiments represented 24% of all experimentally assessed characterization parameters. Thus, without further detailed quantitative assessment, it can be stated broadly that up to 24% of the PCS study parameters could be saved by applying the above-discussed approach, instead of using additional resources. This cost savings, along with the data-driven and graphical justification of control, combine to form a powerful tool to both reduce costs and simultaneously increase process understanding—normally a paradigm that has the opposite correlation.

## 5. Conclusions

Leveraging development data to create in silico IPM models improves upon current best practices by enabling faster establishment of QbD deliverables of risk assessment rankings and preliminary control strategies, ultimately leading to less future experimental effort based on better understanding of the available data, thus leading to significantly better process understanding and control.

A promising next step in this research would be to attempt to automate other standard FMEA rankings (e.g., occurrence or detectability) using an adjusted concept. For example, it could be possible to simulate occurrence results by estimating the capability values of the IPM simulated distributions. The ultimate goal in this work would be to generate the entire FMEA by using the IPM in development.

These approaches are limited in that they require the presence of data. Further research may therefore investigate the establishment of a multiple-product encompassing ‘platform’ IPM, which can serve as a starting point for the first-iteration FMEAs, which could point to the most probable CPPs based on knowledge from previous projects.

For future applications such as process monitoring, the IPM can be updated with new in-process manufacturing data. The IPM can then be used to predict the out-of-specification probability of the currently running campaign. If this probability is undesirably high, the model can then be used to propose changes in process parameter set points to lower the out-of-specification probability to acceptable levels. This constant update with data and feedback into the system could turn the IPM into a category of digital twin [21].

With the continuous exploration of advanced in silico process models, development data should increasingly be seen as a vital basis for IPMs. The technology presented here fully demonstrates the power of applying statistical tools to maximize the knowledge gained from the available data and how focused and efficient knowledge management can be used to invert the paradigm of increased process understanding being associated with increased development costs.

## Figures and Tables

**Table 1:** Summary of models used for Monte Carlo simulation. DoE models are multiple linear regression models from statistically underpinned designs. SC models are single-factor linear regression models of load to specific clearance. If both types of model were available, ‘Both’ is marked in the table.

Unit Operation	CQA1_imp_	CQA2_imp_	CQA3_imp_	CQA1_product_
CAP	DoE	DoE		DoE
AT	Both	DoE	DoE	DoE
DF			SC Model	
AEX	DoE	Both	Both	DoE
CEX	Both	DoE	DoE	DoE
VF				
UFDF	SC Model	SC Model		

**Table 2:** FMEA ranking algorithm.

% Reference	FMEA Ranking
≥0.8	10
0.5–0.8	7
0.3–0.5	3
<0.3	1

**Table 3:** Summary overview of automated results for PAR setting by PSA.

Unit Operation	Process Parameter	Proposed Control Range (If Any)	Justification of PAR
AT	pH AT	Restriction Low	CQA4_prod_, CQA1_imp_, CQA3_imp_
CEX	Conductivity	Restriction High	CQA1_imp_, CQA2_imp_, CQA3_imp_
	Load Density	Restriction High	CQA1_imp_

## Data Availability

Not applicable.

## Supplementary Materials

The following are available online at https://www.mdpi.com/article/10.3390/bioengineering8110156/s1. Table S1: Overview of identified models based on DoE data. Table S2: Overview of specific clearance models. Figures S1–S3: Simulation trend plots. Figures S4–S6: PSA CQA plots. Figures S7–S9: PSA CPP plots.
