# Supplementary material for: Integrated Process Model Applications Linking Bioprocess Development to Quality by Design Milestones
Source: Bioengineering (Basel). 2021 Oct 24;8(11):156. doi: 10.3390/bioengineering8110156 (PMC8614990; doi:10.3390/bioengineering8110156)
Supplement: Supplementary file 1 [file bioengineering-08-00156-s001.zip › bioengineering-1395381-supplementary.pdf]

Supplemental Data and Plots

Linking Bioprocess Development to Quality-by-Design Milestones via Digital Twin Applications

Model tables

Table S1: Overview of identified models based on DoE data. AIC was used as a primary identifier of selected model. R^2, Q^2, RMSE, and p-values, as well as residual analysis, were used alongside process expertise to determine acceptance of the model within the IPM. All residual variation is accounted for within the prediction interval of the simulated values within the IPM. (+) indicates positive coefficient and (-) indicates negative coefficient.

Unit Op	Blinded	Response	Model	R^2	Q^2	RMSE	P	Parameters
CAP	$CQA1_{prod}$	Main SCX	Quadratic	0.45	0.34	1.119	0.019	(-) Load Density<br>(+) Load Density^2<br>(-) Residence Time
	$CQA1_{imp}$	BPG	Linear	0.24	0.11	0.229	0.011	(+) Residence Time
	$CQA2_{imp}$	HMW SEC	Quadratic	0.67	0.57	0.309	0.0001	(-) Load Density<br>(+) Load Density^2
AT	$CQA1_{prod}$	Main SCX	Interaction/Quadratic	0.85	0.55	0.016	0.0001	(-) Concentration<br>(+) pH AT<br>(-) Hold Time AT<br>(+) Concentration * pH AT<br>(-) Concentration * Hold AT<br>(+) pH AT * Hold AT<br>(-) pH AT^2
	$CQA1_{imp}$	BPG	Interaction/Quadratic	0.94	0.92	0.04	0.0001	(-) Concentration<br>(+) pH AT<br>(-) Hold Time AT<br>(+) Neutr. pH<br>(+) Concentration * pH AT<br>(-) Concentration * Neutr. pH<br>(+) pH AT * Hold AT<br>(-) pH AT^2<br>(+) Hold AT^2
	$CQA2_{imp}$	HMW SEC	Interaction/Quadratic	0.92	0.89	0.672	0.0001	(-) Concentration<br>(+) pH AT<br>(-) Hold Time AT<br>(+) Neutr. Hold Time<br>(-) pH AT * Neutr. Hold<br>(+) Concentration^2<br>(+) Hold AT^2<br>(+) Neutr. Hold ^{2}
	$CQA3_{imp}$	LMW SEC	Interaction/Quadratic	0.43	0.12	0.031	0.0087	(-) Concentration<br>(+) pH AT<br>(-) Hold Time AT<br>(+) Neutr. Hold Time<br>(-) Neutr. pH<br>(-) Concentration * pH AT<br>(-) Hold AT^2<br>(+) Neutr. pH^2<br>(-) Concentration^2

Supplemental Data and Plots

Linking Bioprocess Development to Quality-by-Design Milestones via Digital Twin Applications

Unit Op	Blinded	Response	Model	R^2	Q^2	RMSE	P	Parameters
AEX	$CQA1_{prod}$	Main SCX	Interaction	0.77	0.68	0.01	0.0001	(-) Load Density<br>(-) Equil. pH<br>(+) Load Density * Equil pH
	$CQA1_{imp}$	BPG	Quadratic	0.53	0.43	0.103	0.002	(-) Equil. pH^2<br>(+) Equil. pH<br>(+) Equil. pH^2
	$CQA2_{imp}$	HMW SEC	Quadratic	0.65	0.17	0.067	0.0001	(+) Equil. pH<br>(-) Equil. Conductivity<br>(+) Equil. pH^2
	$CQA3_{imp}$	LMW SEC	Quadratic	0.54	0.41	0.037	0.0005	(+) Equil. pH<br>(-) Equil. Conductivity<br>(+) Equil. $Conductivity^2$
CEX	$CQA1_{prod}$	Main SCX	Interaction / Quadratic	0.76	0.64	0.001	0.0001	(+) Elu. Conductivity<br>(+) Load Density<br>(+) Load Conductivity<br>(+) Load / Elu. pH<br>(-) Elu. Cond. * Load / Elu. pH<br>(+) Load Cond. ^{2}<br>(-) Load / Elu. pH^2
	$CQA1_{imp}$	BPG	Interaction / Quadratic	0.77	0.65	0.068	0.0001	(-) Elu. Conductivity<br>(-) Load Density<br>(-) Load / Elu. pH<br>(+) Elu. Cond. * Load Density<br>(+) Elu. Cond. * Load / Elu. pH<br>(+) Elu $Cond.^2$
	$CQA2_{imp}$	HMW SEC	Linear	0.33	0.22	0.598	0.0005	(-) Elu. Conductivity<br>(-) Load / Elu. pH
	$CQA3_{imp}$	LMW SEC	Quadratic	0.4	0.31	0.434	0.0007	(-) Elu. Conductivity<br>(+) Load Conductivity<br>(+) Load / Elu. pH<br>(-) Load $Cond.^2$

Supplemental Data and Plots

Linking Bioprocess Development to Quality-by-Design Milestones via Digital Twin Applications

Table S2: Overview of models showing a correlation between specific CQA clearances and CQA load (load models). (+) indicates a positive regression line between clearance and load, whereas (-) indicates a negative regression line between clearance and load

Unit Operation	CQA	R^2	Effect
AT	$CQA1_{imp}$	0.75	(+)
AEX	$CQA2_{imp}$	0.76	(+)
	$CQA3_{imp}$	0.70	(-)
DF	$CQA3_{imp}$	0.76	(+)
CEX	$CQA1_{imp}$	0.73	(-)
UFDF	$CQA1_{imp}$	0.95	(-)
	$CQA2_{imp}$	0.95	(+)

Supplemental Data and Plots

Linking Bioprocess Development to Quality-by-Design Milestones via Digital Twin Applications

Simulation Trend Plots

For all plots below, 1000 runs were simulated over the full process(top) and plotted against the sum of existing real manufacturing data (bottom) as well as the Out-of-Acceptance limit (red). Additionally, given only this information, a PPK result is given for OOA, that is, how likely given the normal distribution around the real and simulated data would be OOA at drug substance, which can be used as a comparative diagnostic of IPM quality.

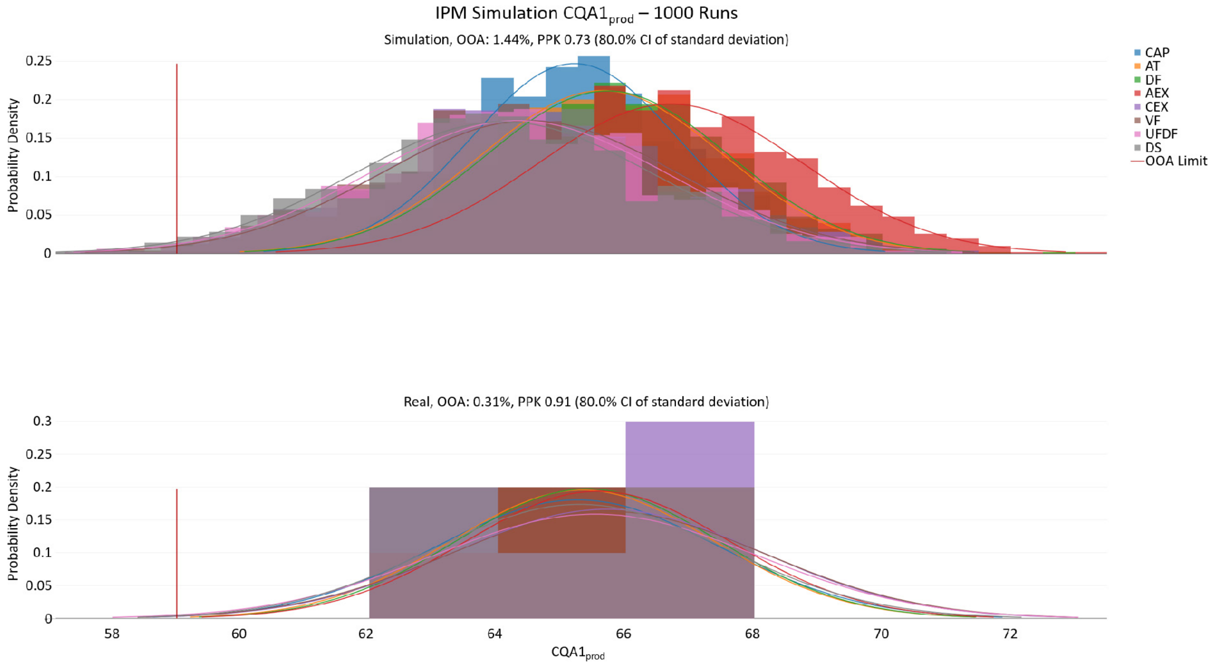

Figure S1: Simulation trend plot for $CQA1_{prod}$

Supplemental Data and Plots

Linking Bioprocess Development to Quality-by-Design Milestones via Digital Twin Applications

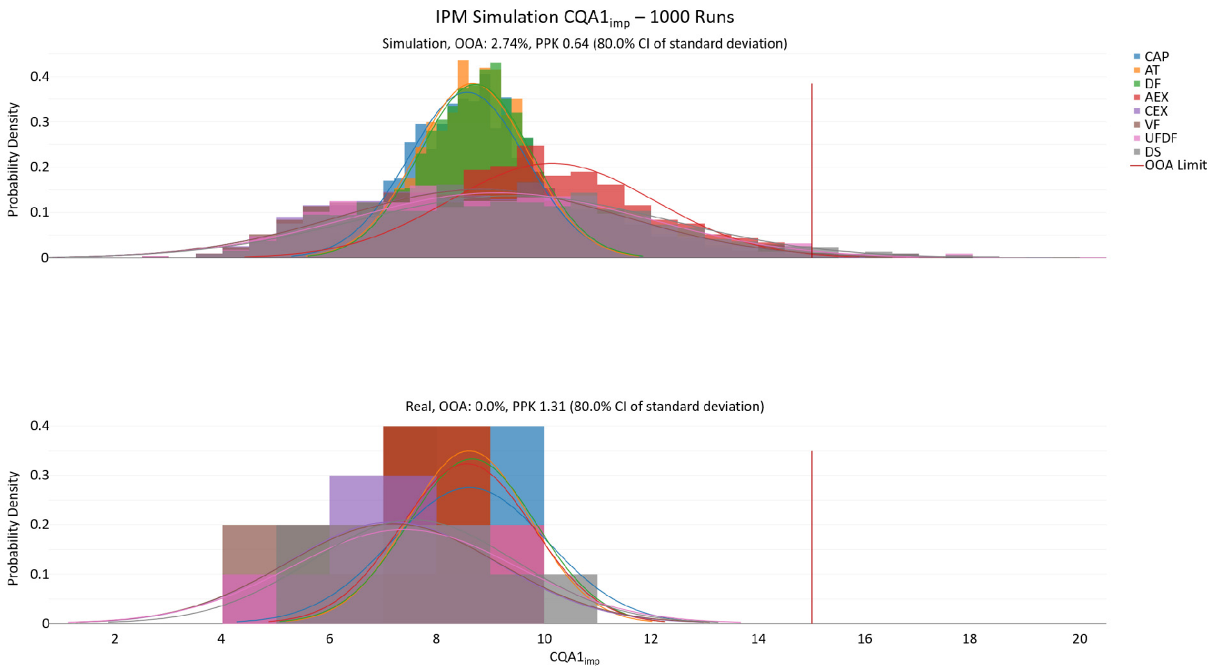

Figure S2: Simulation trend plot for CQA1$_{imp}$

Supplemental Data and Plots

Linking Bioprocess Development to Quality-by-Design Milestones via Digital Twin Applications

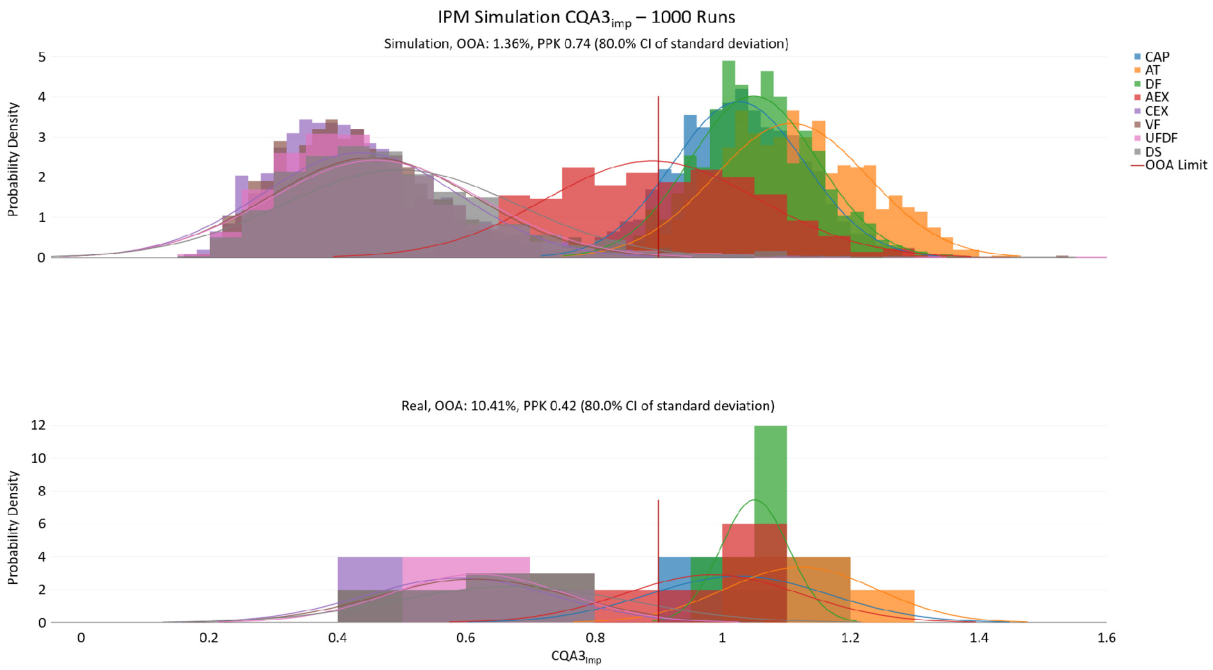

Figure S3: Simulation trend plot for $CQA3_{imp}$

Supplemental Data and Plots

Linking Bioprocess Development to Quality-by-Design Milestones via Digital Twin Applications

PSA CQA Plots

For all plots below, 1000 simulations per grid point were performed over the full process. The results in OOA are plotted across the relative screening range. The screening range is depicted such that 0 is always the set point condition and the remaining screening ranges are coded between -2, 2.

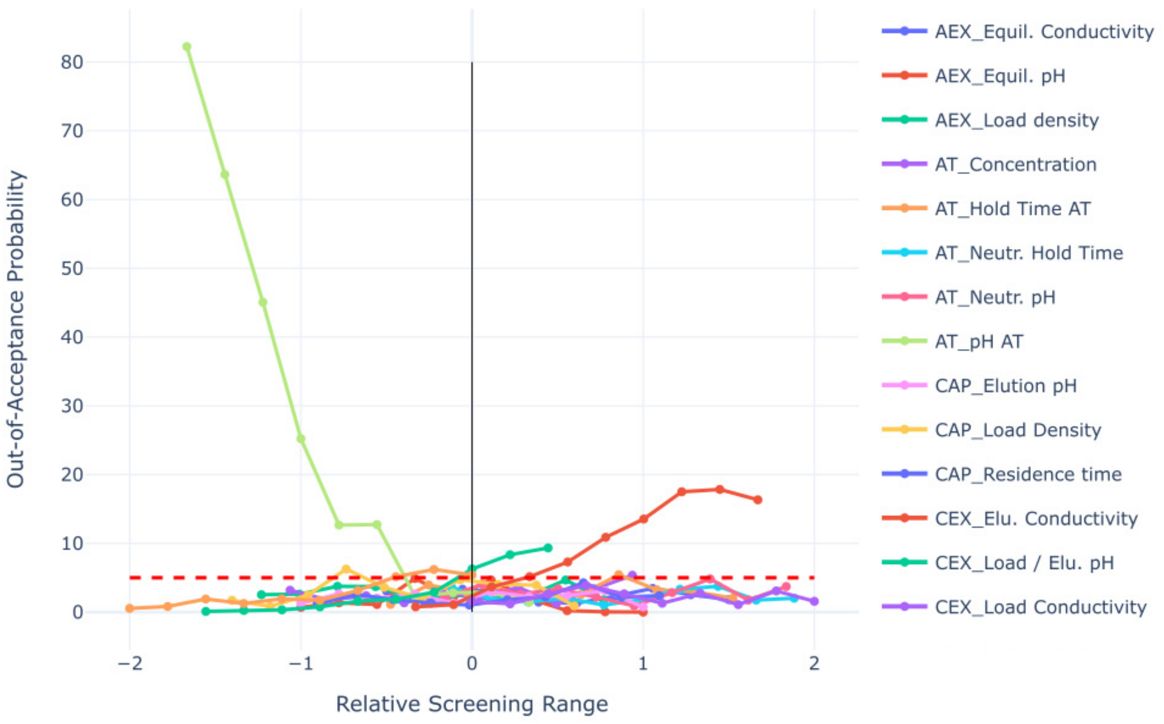

Figure S4: $CQA1_{imp}$ CQA PSA plot

Supplemental Data and Plots

Linking Bioprocess Development to Quality-by-Design Milestones via Digital Twin Applications

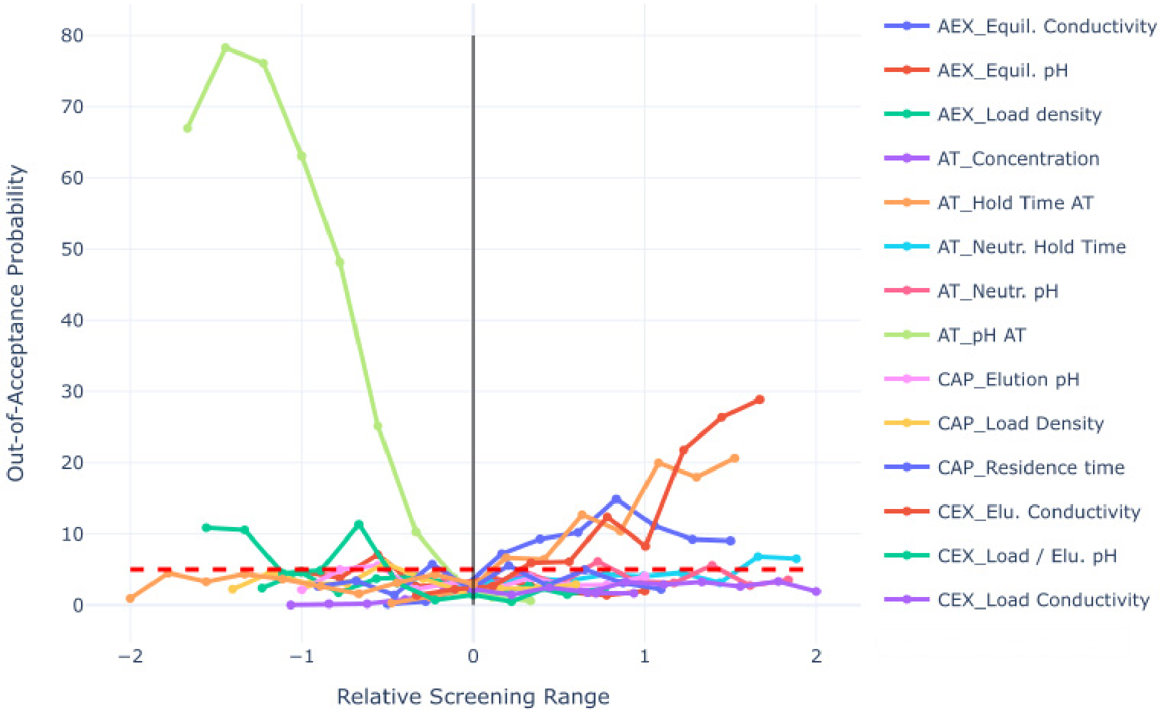

Figure S5: $CQA2_{imp}$ CQA PSA plot

Supplemental Data and Plots

Linking Bioprocess Development to Quality-by-Design Milestones via Digital Twin Applications

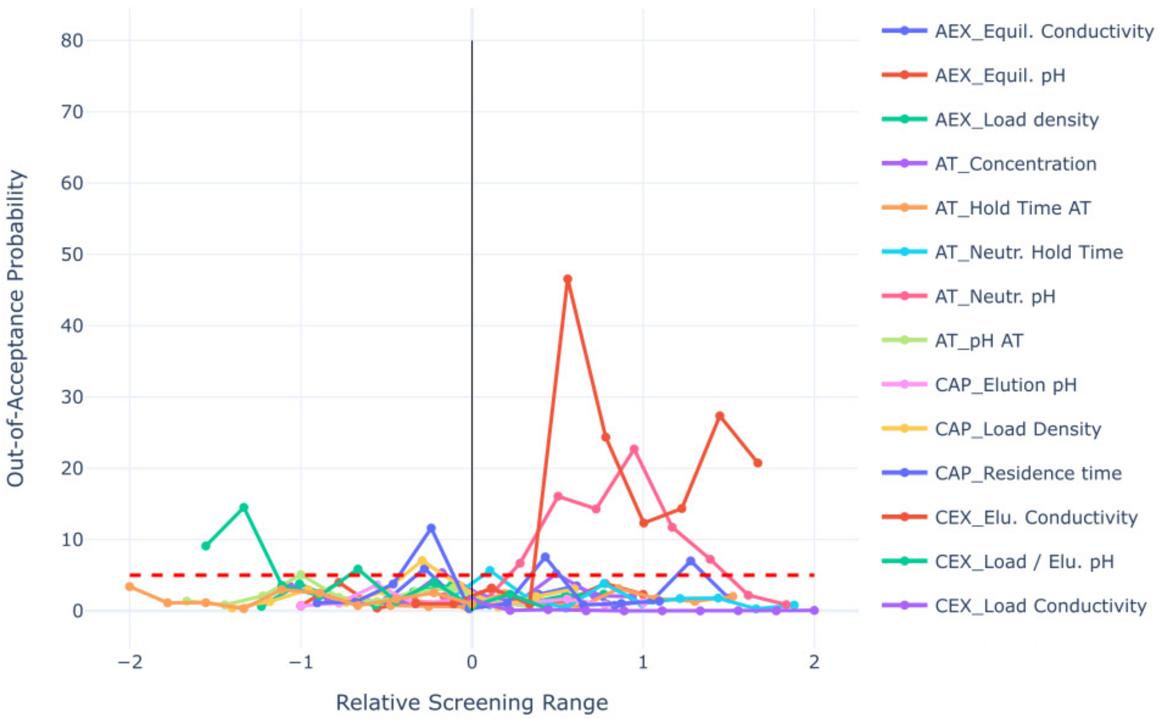

Figure S6: CQA3$_{imp}$ CQA PSA plot

Supplemental Data and Plots

Linking Bioprocess Development to Quality-by-Design Milestones via Digital Twin Applications

PSA CPP Plots

For all plots below, 1000 simulations per grid point were performed over the full process. The results in OOA are plotted across the individual CPP range. The area is grey is excluded from the proposed manufacturing PAR range.

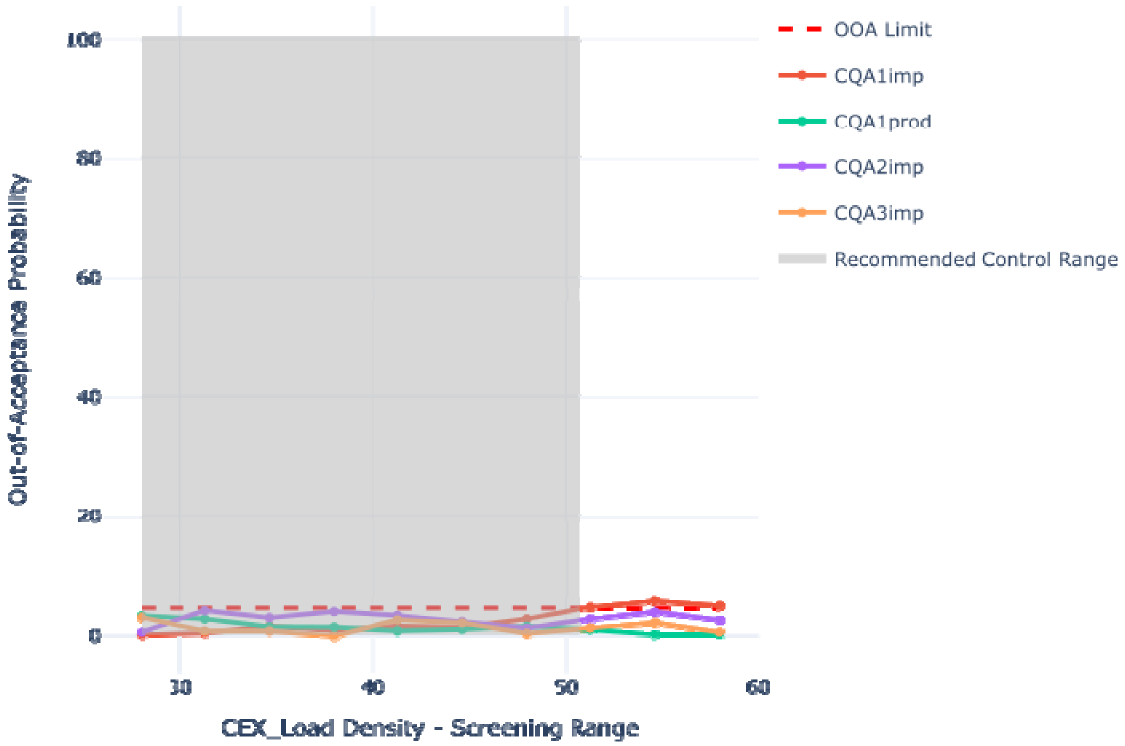

Figure S7: CEX_Load Density CPP PSA plot

Supplemental Data and Plots

Linking Bioprocess Development to Quality-by-Design Milestones via Digital Twin Applications

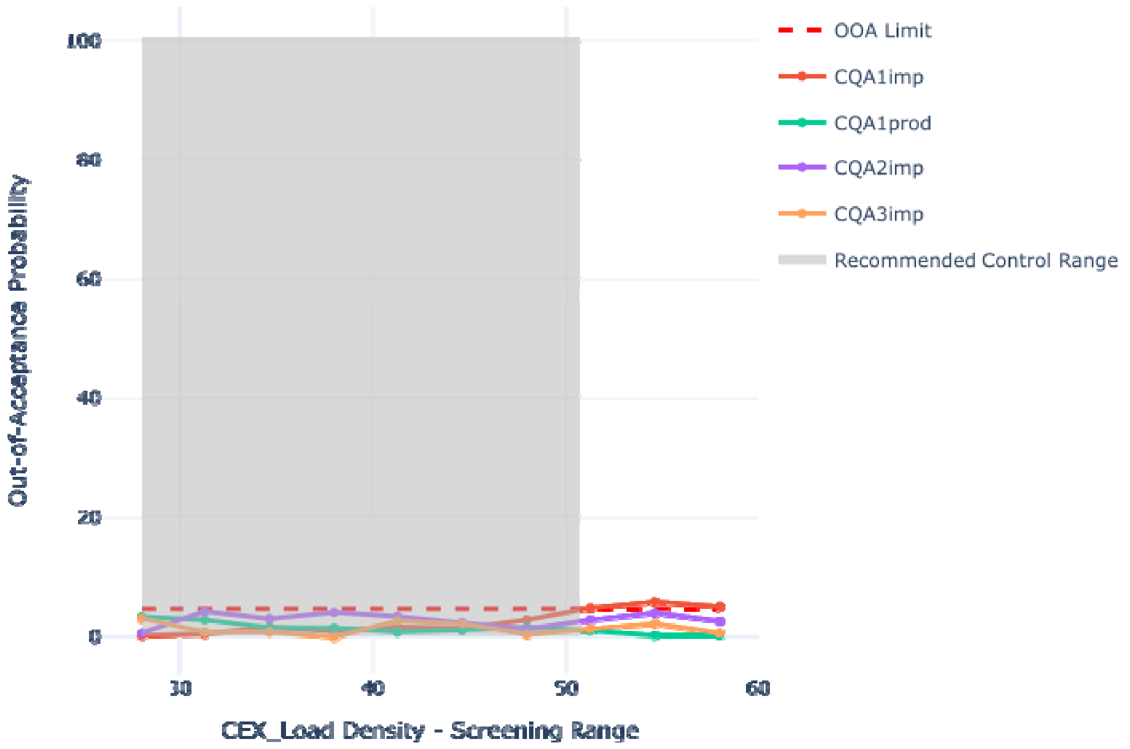

Figure S8: CEX_Load Density CPP PSA Plot

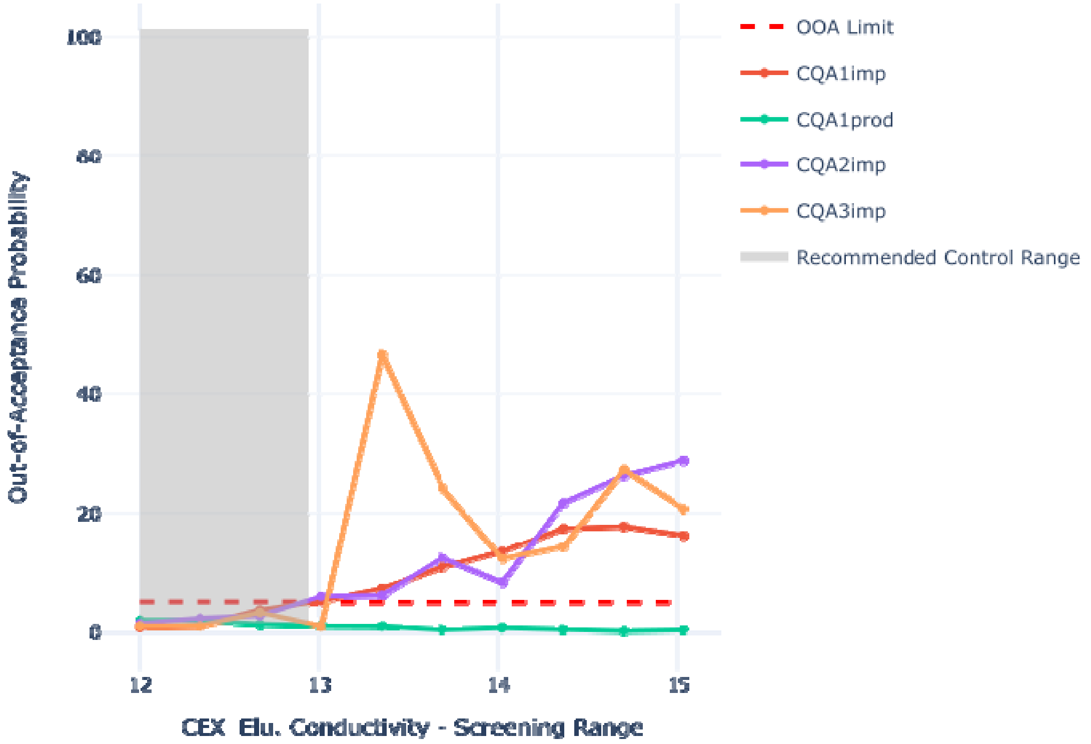

Figure S9: CEX_Elu. Conductivity CPP PSA plot
